# Unusual actinomycosis of the chest wall

**DOI:** 10.11604/pamj.2014.17.61.3368

**Published:** 2014-01-26

**Authors:** Mouna Bouaddi, Badredine Hassam

**Affiliations:** 1 Department of Dermatology and Venereology, university of Medicine, University Mohammed V Souissi, Rabat, Morocco

**Keywords:** Actinomycosis, chest wall, granulomatous infection

## Image in medicine

Actinomycosis is a chronic infection due to anaerobic filamentous bacteria (Actinomyces), which occurs mostly in the cervicofacial region as a chronic granulomatous infection. We report the case of Mr KI, 17 years old, without past medical history, presented a painful ulceration on the right side of the chest wall which evolved since 2 months. The onset of symptoms was a nodule which ulcerated after 2 months with extruding yellowish pus. The examination objectified ulcerated and burgeoning lesion in right parasternal region from what came yellowish pus mixed with sulphur granule (A). A painful subcutaneous nodule was noted under the right breast. An examination of the oral mucosa was entirely normal as well as the rest of the physical examination. Histopathologic studies (hematoxylin-eosin staining) revealed the presence of inflammatory cells surrounding basophilic sulfur granules which were composed of a large number of filaments arranged in a radiating pattern (B). The chest x-ray was normal. The chest scan showed an infiltration of the anterior and right lateral wall without bone and lung injury. The results of aerobic and anaerobic culture showed mixed flora. The histopathologic findings led to the diagnosis of actinomycosis of the wall chest. Treatment was based on oral amoxicillin + clavulanic acid (1g + 125 mg 3 times daily). The size of the ulcerative lesion gradually decreased and completely healed by 4 weeks. Then antibiotherapy was continued for four months. No evidence of recurrence of this lesion was found after 24-month of follow-up.

**Figure 1:**
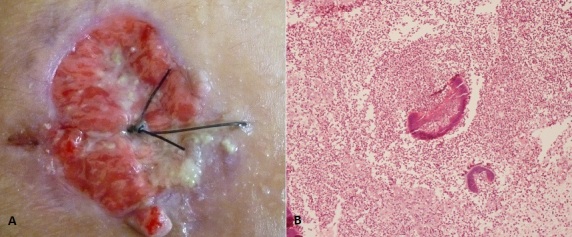
A) ulcerated and burgeoning lesion in right parasternal region from what came yellowish pus mixed with sulphur granule; B)presence of inflammatory cells surrounding basophilic sulfur granules which were composed of a large number of filaments arranged in a radiating pattern

